# Supplementary material for: Projections of human papillomavirus (HPV) vaccination impact on non-cervical cancer outcomes among women in 117 low- and middle-income countries: a modelling study
Source: BMJ Glob Health. 2026 Aug 31;11(8):e021127. doi: 10.1136/bmjgh-2025-021127 (PMC13536154; doi:10.1136/bmjgh-2025-021127)
Supplement: Supplementary file 1 [file bmjgh-11-8-s001.pdf]

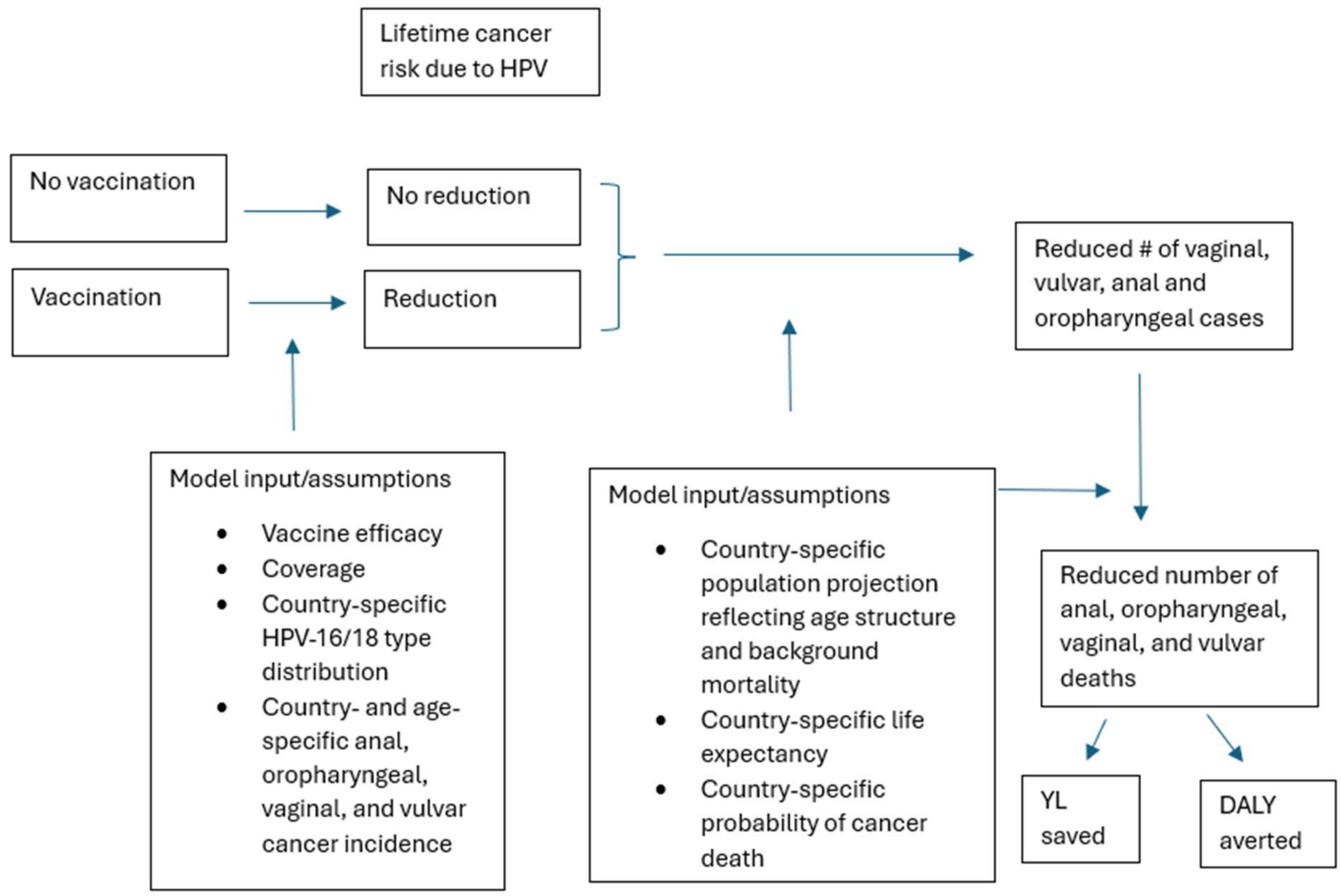

Figure S1: An overview of the model framework.

Note: YL refers to Years of Life and DALY refers to Disability-Adjusted Life Years.
