# Supplementary material for: Projections of human papillomavirus (HPV) vaccination impact on non-cervical cancer outcomes among women in 117 low- and middle-income countries: a modelling study
Source: BMJ Glob Health. 2026 Aug 31;11(8):e021127. doi: 10.1136/bmjgh-2025-021127 (PMC13536154; doi:10.1136/bmjgh-2025-021127)
Supplement: Supplementary file 3 [file bmjgh-11-8-s003.pdf]

APPENDIX

Parameter	Description of Assumption
Cohort Structure	The assumption is that girls fully immunized at a target age and tracked through their lifetimes. The model assumes full vaccination and lifelong immunity.
HPV Type Distribution	HPV-16/18 types are attributed to cervical cancer cases.
Vaccine Cross-Protection	There is no cross-protection assumed against non-vaccine HPV types.
Probabilistic Sensitivity Analysis (PSA)	Parameters are sampled using b-PERT distribution with bounds based on empirical data and assumptions. There are 200 iterations per country.

Table S1: Summary of key model assumptions.

Country	Region, Income Category	Matched Country	Cancer Type
Grenada	AMR, umi	Guatemala	Oropharyngeal
Kiribati	WPR, lmi	Cambodia	Anal, oropharyngeal, vaginal, and vulvar
Marshall Islands	WPR, umi	China	Oropharyngeal, vaginal, and vulvar
Tonga	WPR, umi	China	Anal, oropharyngeal, vaginal, and vulvar
Tuvalu	WPR, umi	China	Anal, oropharyngeal, vaginal, and vulvar
St Vincent & the Grenadines	AMR, umi	Ecuador	Anal, oropharyngeal, vaginal, and vulvar
Grenada	AMR, umi	Colombia	Anal, vaginal, vulvar
Palestine	EMR, lmi	Egypt	Anal, oropharyngeal, vaginal, and vulvar
Kosovo	EUR	Serbia (neighboring country)	Anal, oropharyngeal, vaginal, and vulvar

Table S2: Region and income-level matched country assumptions for countries with missing data for age-specific incidence rate by cancer type.

Note: AMR= Region of the Americas; EMR= Eastern Mediterranean Region; EUR= European Region; lmi= lower-middle income; umi= upper-middle income; WPR= Western Pacific Region.

Country Name	Region	Income Level	Type distribution mean	Lower Bound	Upper Bound	Source
Afghanistan	EMR	low	0.9	0.85	0.95	16 (SEAR average) *
Angola	AFR	lmi	0.78	0.73	0.83	16
Albania	EUR	umi	0.91	0.86	0.96	16
Armenia	EUR	umi	0.91	0.86	0.96	16
Azerbaijan	EUR	umi	0.91	0.86	0.96	16
Burundi	AFR	low	0.78	0.73	0.83	16
Benin	AFR	lmi	0.78	0.73	0.83	16
Burkina Faso	AFR	low	0.78	0.73	0.83	16
Bangladesh	SEAR	lmi	0.9	0.85	0.95	16
Bosnia and Herzegovina	EUR	umi	0.91	0.86	0.96	16
Belarus	EUR	umi	0.91	0.86	0.96	16
Belize	AMR	umi	0.84	0.79	0.89	16
Bolivia, Plurinational State of	AMR	lmi	0.84	0.79	0.89	16
Bhutan	SEAR	lmi	0.9	0.85	0.95	16
Central African Republic	AFR	low	0.78	0.73	0.83	16
China (Asia)	WPR	umi	0.9	0.85	0.95	16
Cote d'Ivoire	AFR	lmi	0.78	0.73	0.83	16
Cameroon	AFR	lmi	0.78	0.73	0.83	16
Congo, the Democratic Republic of the	AFR	low	0.78	0.73	0.83	16
Congo	AFR	lmi	0.78	0.73	0.83	16
Colombia	AMR	umi	0.84	0.79	0.89	16
Comoros	AFR	lmi	0.78	0.73	0.83	16
Cabo Verde	AFR	lmi	0.78	0.73	0.83	16
Cuba	AMR	umi	0.84	0.79	0.89	16
Djibouti	EMR	lmi	0.78	0.73	0.83	16 (AFR average) *
Dominica	AMR	umi	0.84	0.79	0.89	16
Algeria	AFR	lmi	0.78	0.73	0.83	16
Ecuador	AMR	umi	0.84	0.79	0.89	16
Egypt	EMR	lmi	0.78	0.73	0.83	16 (AFR average) *
Eritrea	AFR	low	0.78	0.73	0.83	16
Ethiopia	AFR	low	0.78	0.73	0.83	16
Fiji	WPR	umi	0.9	0.85	0.95	16

Micronesia, Federated States of	WPR	lmi	0.9	0.85	0.95	16
Georgia	EUR	umi	0.91	0.86	0.96	16
Ghana	AFR	lmi	0.78	0.73	0.83	16
Guinea	AFR	lmi	0.78	0.73	0.83	16
Gambia	AFR	low	0.78	0.73	0.83	16
Guinea-Bissau	AFR	low	0.78	0.73	0.83	16
Grenada	AMR	umi	0.84	0.79	0.89	16
Guatemala	AMR	umi	0.84	0.79	0.89	16
Guyana	AMR		0.84	0.79	0.89	16
Honduras	AMR	lmi	0.84	0.79	0.89	16
Haiti	AMR	lmi	0.84	0.79	0.89	16
Indonesia	SEAR	umi	0.9	0.85	0.95	16
India	SEAR	lmi	0.9	0.85	0.95	16
Iran, Islamic Republic of	EMR	lmi	0.9	0.85	0.95	16 (SEAR average) *
Iraq	EMR	umi	0.9	0.85	0.95	16 (SEAR average) *
Jamaica	AMR	umi	0.84	0.79	0.89	16
Jordan	EMR	lmi	0.9	0.85	0.95	16
Kenya	AFR	lmi	0.78	0.73	0.83	16
Kyrgyzstan	EUR		0.91	0.86	0.96	16
Cambodia	WPR	lmi	0.9	0.85	0.95	16
Kiribati	WPR	lmi	0.9	0.85	0.95	16
Lao People's Democratic Republic	WPR	lmi	0.9	0.85	0.95	16
Liberia	AFR	low	0.78	0.73	0.83	16
St. Lucia	AMR	umi	0.84	0.79	0.89	16
Sri Lanka	SEAR	lmi	0.9	0.85	0.95	16
Lesotho	AFR	lmi	0.78	0.73	0.83	16
Morocco	EMR	lmi	0.78	0.73	0.83	16 (AFR average) *
Moldova, Republic of	EUR	umi	0.91	0.86	0.96	16
Madagascar	AFR	low	0.78	0.73	0.83	16
Maldives	SEAR	umi	0.9	0.85	0.95	16
Marshall Islands	WPR	umi	0.9	0.85	0.95	16
Macedonia, the former Yugoslav Republic of	EUR		0.91	0.86	0.96	16
Mali	AFR	low	0.78	0.73	0.83	16

Myanmar	SEAR	lmi	0.9	0.85	0.95	16
Mongolia	WPR	lmi	0.9	0.85	0.95	16
Mozambique	AFR	low	0.78	0.73	0.83	16
Mauritania	AFR	lmi	0.78	0.73	0.83	16
Malawi	AFR	low	0.78	0.73	0.83	16
Namibia	AFR	umi	0.78	0.73	0.83	16
Niger	AFR	low	0.78	0.73	0.83	16
Nigeria	AFR	lmi	0.78	0.73	0.83	16
Nicaragua	AMR	lmi	0.84	0.79	0.89	16
Nepal	SEAR	lmi	0.9	0.85	0.95	16
Pakistan	EMR	lmi	0.9	0.85	0.95	16 (SEAR average) *
Peru	AMR	umi	0.84	0.79	0.89	16
Philippines (Asia)	WPR	lmi	0.9	0.85	0.95	16
Papua New Guinea	WPR	lmi	0.9	0.85	0.95	16
Korea, Democratic People's Republic of	SEAR	low	0.9	0.85	0.95	16
Paraguay	AMR	umi	0.84	0.79	0.89	16
Palestine, State of	EMR	lmi	0.9	0.85	0.95	16 (SEAR average) *
Rwanda	AFR	low	0.78	0.73	0.83	16
Sudan	EMR	low	0.78	0.73	0.83	16 (AFR average) *
Senegal	AFR	lmi	0.78	0.73	0.83	16
Solomon Islands	WPR	lmi	0.9	0.85	0.95	16
Sierra Leone	AFR	low	0.78	0.73	0.83	16
El Salvador	AMR	umi	0.84	0.79	0.89	16
Somalia	EMR	low	0.78	0.73	0.83	16 (AFR average) *
Serbia	EUR	umi	0.91	0.86	0.96	16
South Sudan	AFR	low	0.78	0.73	0.83	16
Sao Tome and Principe	AFR	lmi	0.78	0.73	0.83	16
Swaziland	AFR		0.78	0.73	0.83	16
Syrian Arab Republic	EMR	low	0.9	0.85	0.95	16 (SEAR average) *
Chad	AFR	low	0.78	0.73	0.83	16
Togo	AFR	low	0.78	0.73	0.83	16
Thailand	SEAR	umi	0.9	0.85	0.95	16 (SEAR average) *
Tajikistan	EUR	lmi	0.91	0.86	0.96	16
Turkmenistan	EUR	umi	0.91	0.86	0.96	16
Timor-Leste	SEAR	lmi	0.9	0.85	0.95	16

Tonga	WPR	umi	0.9	0.85	0.95	16
Tunisia	EMR	lmi	0.78	0.73	0.83	16 (AFR average) *
Tuvalu	WPR	umi	0.9	0.85	0.95	16
Tanzania, United Republic of	AFR	lmi	0.78	0.73	0.83	16
Uganda	AFR	low	0.78	0.73	0.83	16
Ukraine	EUR	lmi	0.91	0.86	0.96	16
Uzbekistan	EUR	lmi	0.91	0.86	0.96	16
St. Vincent & the Grenadines	AMR	umi	0.84	0.79	0.89	16
Venezuela, Bolivarian Republic of	AMR		0.84	0.79	0.89	16
Viet Nam	WPR	lmi	0.9	0.85	0.95	16
Vanuatu	WPR	lmi	0.9	0.85	0.95	16
Samoa	WPR		0.9	0.85	0.95	16
Kosovo	EUR	umi	0.91	0.86	0.96	16
Yemen	EMR	low	0.9	0.85	0.95	16 (SEAR average) *
South Africa	AFR	umi	0.78	0.73	0.83	16
Zambia	AFR	lmi	0.78	0.73	0.83	16
Zimbabwe	AFR	lmi	0.78	0.73	0.83	16

Table S3: Type distribution assumptions for anal cancer.

Note: Asterisks (*) denote instances where the authors performed calculations to determine the average. AFR= African Region; AMR= Region of the Americas; EMR= Eastern Mediterranean Region; EUR= European Region; lb= lower bound; lmi= lower-middle income; ub= upper bound; umi= upper-middle income; WPR= Western Pacific Region.

Country Name	Region	Income Level	Type distribution mean	Lower Bound	Upper Bound	Source
Afghanistan	EMR	low	0.95	0.19	1	16 (SEAR average) *
Angola	AFR	lmi	0.13	0	0.25	17, 18
Albania	EUR	umi	0.92	0.16	1	16
Armenia	EUR	umi	0.92	0.16	1	16
Azerbaijan	EUR	umi	0.92	0.16	1	16
Burundi	AFR	low	0.13	0	0.25	17, 18
Benin	AFR	lmi	0.13	0	0.25	17, 18
Burkina Faso	AFR	low	0.13	0	0.25	17, 18
Bangladesh	SEAR	lmi	0.95	0.19	1	16
Bosnia and Herzegovina	EUR	umi	0.92	0.16	1	16
Belarus	EUR	umi	0.92	0.16	1	16
Belize	AMR	umi	0.81	0.05	1	16
Bolivia, Plurinational State of	AMR	lmi	0.81	0.05	1	16
Bhutan	SEAR	lmi	0.95	0.19	1	16
Central African Republic	AFR	low	0.13	0	0.25	17, 18
China (Asia)	WPR	umi	0.95	0.19	1	16
Cote d'Ivoire	AFR	lmi	0.13	0	0.25	17, 18
Cameroon	AFR	lmi	0.13	0	0.25	17, 18
Congo, the Democratic Republic of the	AFR	low	0.13	0	0.25	17, 18
Congo	AFR	lmi	0.13	0	0.25	17, 18
Colombia	AMR	umi	0.81	0.05	1	16
Comoros	AFR	lmi	0.13	0	0.25	17, 18
Cabo Verde	AFR	lmi	0.13	0	0.25	17, 18
Cuba	AMR	umi	0.81	0.05	1	16
Djibouti	EMR	lmi	0.13	0	0.25	17, 18 (AFR average) *
Dominica	AMR	umi	0.81	0.05	1	16
Algeria	AFR	lmi	0.13	0	0.25	17, 18
Ecuador	AMR	umi	0.81	0.05	1	16
Egypt	EMR	lmi	0.13	0	0.25	17, 18 (AFR average) *
Eritrea	AFR	low	0.13	0	0.25	17, 18
Ethiopia	AFR	low	0.13	0	0.25	17, 18
Fiji	WPR	umi	0.95	0.19	1	16
Micronesia, Federated States of	WPR	lmi	0.95	0.19	1	16

Georgia	EUR	umi	0.92	0.16	1	16
Ghana	AFR	lmi	0.13	0	0.25	17, 18
Guinea	AFR	lmi	0.13	0	0.25	17, 18
Gambia	AFR	low	0.13	0	0.25	17, 18
Guinea-Bissau	AFR	low	0.13	0	0.25	17, 18
Grenada	AMR	umi	0.81	0.05	1	16
Guatemala	AMR	umi	0.81	0.05	1	16
Guyana	AMR		0.81	0.05	1	16
Honduras	AMR	lmi	0.81	0.05	1	16
Haiti	AMR	lmi	0.81	0.05	1	16
Indonesia	SEAR	umi	0.95	0.19	1	16
India	SEAR	lmi	0.95	0.19	1	16
Iran, Islamic Republic of	EMR	lmi	0.95	0.19	1	16 (SEAR average) *
Iraq	EMR	umi	0.95	0.19	1	16 (SEAR average) *
Jamaica	AMR	umi	0.81	0.05	1	16
Jordan	EMR	lmi	0.95	0.19	1	16
Kenya	AFR	lmi	0.13	0	0.25	17, 18
Kyrgyzstan	EUR		0.92	0.16	1	16
Cambodia	WPR	lmi	0.95	0.19	1	16
Kiribati	WPR	lmi	0.95	0.19	1	16
Lao People's Democratic Republic	WPR	lmi	0.95	0.19	1	16
Liberia	AFR	low	0.13	0	0.25	17, 18
St. Lucia	AMR	umi	0.81	0.05	1	16
Sri Lanka	SEAR	lmi	0.95	0.19	1	16
Lesotho	AFR	lmi	0.13	0	0.25	17, 18
Morocco	EMR	lmi	0.13	0	0.25	17, 18 (AFR average) *
Moldova, Republic of	EUR	umi	0.92	0.16	1	16
Madagascar	AFR	low	0.13	0	0.25	17, 18
Maldives	SEAR	umi	0.95	0.19	1	16
Marshall Islands	WPR	umi	0.95	0.19	1	16
Macedonia, the former Yugoslav Republic of	EUR		0.92	0.16	1	16
Mali	AFR	low	0.13	0	0.25	17, 18
Myanmar	SEAR	lmi	0.95	0.19	1	16
Mongolia	WPR	lmi	0.95	0.19	1	16
Mozambique	AFR	low	0.13	0	0.25	17, 18
Mauritania	AFR	lmi	0.13	0	0.25	17, 18
Malawi	AFR	low	0.13	0	0.25	17, 18

Namibia	AFR	umi	0.13	0	0.25	17, 18
Niger	AFR	low	0.13	0	0.25	17, 18
Nigeria	AFR	lmi	0.13	0	0.25	17, 18
Nicaragua	AMR	lmi	0.81	0.05	1	16
Nepal	SEAR	lmi	0.95	0.19	1	16
Pakistan	EMR	lmi	0.95	0.19	1	16 (SEAR average) *
Peru	AMR	umi	0.81	0.05	1	16
Philippines (Asia)	WPR	lmi	0.95	0.19	1	16
Papua New Guinea	WPR	lmi	0.95	0.19	1	16
Korea, Democratic People's Republic of	SEAR	low	0.95	0.19	1	16
Paraguay	AMR	umi	0.81	0.05	1	16
Palestine, State of	EMR	lmi	0.95	0.19	1	16 (SEAR average) *
Rwanda	AFR	low	0.13	0	0.25	17, 18
Sudan	EMR	low	0.13	0	0.25	17,18 (AFR average) *
Senegal	AFR	lmi	0.13	0	0.25	17, 18
Solomon Islands	WPR	lmi	0.95	0.19	1	16
Sierra Leone	AFR	low	0.13	0	0.25	17, 18
El Salvador	AMR	umi	0.81	0.05	1	16
Somalia	EMR	low	0.13	0	0.25	17, 18 (AFR average) *
Serbia	EUR	umi	0.92	0.16	1	16
South Sudan	AFR	low	0.13	0	0.25	17, 18
Sao Tome and Principe	AFR	lmi	0.13	0	0.25	17, 18
Swaziland	AFR		0.13	0	0.25	17, 18
Syrian Arab Republic	EMR	low	0.95	0.19	1	16 (SEAR average) *
Chad	AFR	low	0.13	0	0.25	17, 18
Togo	AFR	low	0.13	0	0.25	17, 18
Thailand	SEAR	umi	0.95	0.19	1	16 (SEAR average) *
Tajikistan	EUR	lmi	0.92	0.16	1	16
Turkmenistan	EUR	umi	0.92	0.16	1	16
Timor-Leste	SEAR	lmi	0.95	0.19	1	16
Tonga	WPR	umi	0.95	0.19	1	16
Tunisia	EMR	lmi	0.13	0	0.25	17, 18 (AFR average) *
Tuvalu	WPR	umi	0.95	0.19	1	16
Tanzania, United Republic of	AFR	lmi	0.13	0	0.25	17, 18
Uganda	AFR	low	0.13	0	0.25	17, 18

Ukraine	EUR	lmi	0.92	0.16	1	16
Uzbekistan	EUR	lmi	0.92	0.16	1	16
St. Vincent & the Grenadines	AMR	umi	0.81	0.05	1	16
Venezuela, Bolivarian Republic of	AMR		0.81	0.05	1	16
Viet Nam	WPR	lmi	0.95	0.19	1	16
Vanuatu	WPR	lmi	0.95	0.19	1	16
Samoa	WPR		0.95	0.19	1	16
Kosovo	EUR	umi	0.92	0.16	1	16
Yemen	EMR	low	0.95	0.19	1	16 (SEAR average) *
South Africa	AFR	umi	0.13	0	0.25	17, 18
Zambia	AFR	lmi	0.13	0	0.25	17, 18
Zimbabwe	AFR	lmi	0.13	0	0.25	17, 18

Table S4: Type distribution assumptions for oropharyngeal cancer.

Note: Asterisks (*) denote instances where the authors performed calculations to determine the average. For sources 17 and 18 the lower bound was assumed to be zero per de Sanjosé, et al. (2019), and the upper bound was set to 25% per Castellsagué, et al. (2016), reflecting HPV-DNA positivity in oropharyngeal cancers globally. AFR= African Region; AMR= Region of the Americas; EMR= Eastern Mediterranean Region; EUR= European Region; lb= lower bound; lmi= lower-middle income; ub= upper bound; umi= upper-middle income; WPR= Western Pacific Region.

Country Name	Region	Income Level	Type distribution mean	Lower Bound	Upper Bound	Source
Afghanistan	EMR	low	0.6	0.48	0.72	16 (SEAR average) *
Angola	AFR	lmi	0.53	0.41	0.65	16
Albania	EUR	umi	0.72	0.60	0.84	16
Armenia	EUR	umi	0.72	0.60	0.84	16
Azerbaijan	EUR	umi	0.72	0.60	0.84	16
Burundi	AFR	low	0.53	0.41	0.65	16
Benin	AFR	lmi	0.53	0.41	0.65	16
Burkina Faso	AFR	low	0.53	0.41	0.65	16
Bangladesh	SEAR	lmi	0.6	0.48	0.72	16
Bosnia and Herzegovina	EUR	umi	0.72	0.60	0.84	16
Belarus	EUR	umi	0.72	0.60	0.84	16
Belize	AMR	umi	0.6	0.48	0.72	16
Bolivia, Plurinational State of	AMR	lmi	0.6	0.48	0.72	16
Bhutan	SEAR	lmi	0.6	0.48	0.72	16
Central African Republic	AFR	low	0.53	0.41	0.65	16
China (Asia)	WPR	umi	0.6	0.48	0.72	16
Cote d'Ivoire	AFR	lmi	0.53	0.41	0.65	16
Cameroon	AFR	lmi	0.53	0.41	0.65	16
Congo, the Democratic Republic of the	AFR	low	0.53	0.41	0.65	16
Congo	AFR	lmi	0.53	0.41	0.65	16
Colombia	AMR	umi	0.6	0.48	0.72	16
Comoros	AFR	lmi	0.53	0.41	0.65	16
Cabo Verde	AFR	lmi	0.53	0.41	0.65	16
Cuba	AMR	umi	0.6	0.48	0.72	16
Djibouti	EMR	lmi	0.53	0.41	0.65	16 (AFR average) *
Dominica	AMR	umi	0.6	0.48	0.72	16
Algeria	AFR	lmi	0.53	0.41	0.65	16
Ecuador	AMR	umi	0.6	0.48	0.72	16
Egypt	EMR	lmi	0.53	0.41	0.65	16 (AFR average) *
Eritrea	AFR	low	0.53	0.41	0.65	16
Ethiopia	AFR	low	0.53	0.41	0.65	16
Fiji	WPR	umi	0.6	0.48	0.72	16

Micronesia, Federated States of	WPR	lmi	0.6	0.48	0.72	16
Georgia	EUR	umi	0.72	0.60	0.84	16
Ghana	AFR	lmi	0.53	0.41	0.65	16
Guinea	AFR	lmi	0.53	0.41	0.65	16
Gambia	AFR	low	0.53	0.41	0.65	16
Guinea-Bissau	AFR	low	0.53	0.41	0.65	16
Grenada	AMR	umi	0.6	0.48	0.72	16
Guatemala	AMR	umi	0.6	0.48	0.72	16
Guyana	AMR		0.6	0.48	0.72	16
Honduras	AMR	lmi	0.6	0.48	0.72	16
Haiti	AMR	lmi	0.6	0.48	0.72	16
Indonesia	SEAR	umi	0.6	0.48	0.72	16
India	SEAR	lmi	0.6	0.48	0.72	16
Iran, Islamic Republic of	EMR	lmi	0.6	0.48	0.72	16 (SEAR average) *
Iraq	EMR	umi	0.6	0.48	0.72	16 (SEAR average) *
Jamaica	AMR	umi	0.6	0.48	0.72	16
Jordan	EMR	lmi	0.6	0.48	0.72	16
Kenya	AFR	lmi	0.53	0.41	0.65	16
Kyrgyzstan	EUR		0.72	0.60	0.84	16
Cambodia	WPR	lmi	0.6	0.48	0.72	16
Kiribati	WPR	lmi	0.6	0.48	0.72	16
Lao People's Democratic Republic	WPR	lmi	0.6	0.48	0.72	16
Liberia	AFR	low	0.53	0.41	0.65	16
St. Lucia	AMR	umi	0.6	0.48	0.72	16
Sri Lanka	SEAR	lmi	0.6	0.48	0.72	16
Lesotho	AFR	lmi	0.53	0.41	0.65	16
Morocco	EMR	lmi	0.53	0.41	0.65	16 (AFR average) *
Moldova, Republic of	EUR	umi	0.72	0.60	0.84	16
Madagascar	AFR	low	0.53	0.41	0.65	16
Maldives	SEAR	umi	0.6	0.48	0.72	16
Marshall Islands	WPR	umi	0.6	0.48	0.72	16
Macedonia, the former Yugoslav Republic of	EUR		0.72	0.60	0.84	16
Mali	AFR	low	0.53	0.41	0.65	16

Myanmar	SEAR	lmi	0.6	0.48	0.72	16
Mongolia	WPR	lmi	0.6	0.48	0.72	16
Mozambique	AFR	low	0.53	0.41	0.65	16
Mauritania	AFR	lmi	0.53	0.41	0.65	16
Malawi	AFR	low	0.53	0.41	0.65	16
Namibia	AFR	umi	0.53	0.41	0.65	16
Niger	AFR	low	0.53	0.41	0.65	16
Nigeria	AFR	lmi	0.53	0.41	0.65	16
Nicaragua	AMR	lmi	0.6	0.48	0.72	16
Nepal	SEAR	lmi	0.6	0.48	0.72	16
Pakistan	EMR	lmi	0.6	0.48	0.72	16 (SEAR average) *
Peru	AMR	umi	0.6	0.48	0.72	16
Philippines (Asia)	WPR	lmi	0.6	0.48	0.72	16
Papua New Guinea	WPR	lmi	0.6	0.48	0.72	16
Korea, Democratic People's Republic of	SEAR	low	0.6	0.48	0.72	16
Paraguay	AMR	umi	0.6	0.48	0.72	16
Palestine, State of	EMR	lmi	0.6	0.48	0.72	16 (SEAR average) *
Rwanda	AFR	low	0.53	0.41	0.65	16
Sudan	EMR	low	0.53	0.41	0.65	16 (AFR average) *
Senegal	AFR	lmi	0.53	0.41	0.65	16
Solomon Islands	WPR	lmi	0.6	0.48	0.72	16
Sierra Leone	AFR	low	0.53	0.41	0.65	16
El Salvador	AMR	umi	0.6	0.48	0.72	16
Somalia	EMR	low	0.53	0.41	0.65	16 (AFR average) *
Serbia	EUR	umi	0.72	0.60	0.84	16
South Sudan	AFR	low	0.53	0.41	0.65	16
Sao Tome and Principe	AFR	lmi	0.53	0.41	0.65	16
Swaziland	AFR		0.53	0.41	0.65	16
Syrian Arab Republic	EMR	low	0.6	0.48	0.72	16 (SEAR average) *
Chad	AFR	low	0.53	0.41	0.65	16
Togo	AFR	low	0.53	0.41	0.65	16
Thailand	SEAR	umi	0.6	0.48	0.72	16 (SEAR average) *
Tajikistan	EUR	lmi	0.72	0.60	0.84	16
Turkmenistan	EUR	umi	0.72	0.60	0.84	16
Timor-Leste	SEAR	lmi	0.6	0.48	0.72	16

Tonga	WPR	umi	0.6	0.48	0.72	16
Tunisia	EMR	lmi	0.53	0.41	0.65	16 (AFR average) *
Tuvalu	WPR	umi	0.6	0.48	0.72	16
Tanzania, United Republic of	AFR	lmi	0.53	0.41	0.65	16
Uganda	AFR	low	0.53	0.41	0.65	16
Ukraine	EUR	lmi	0.72	0.60	0.84	16
Uzbekistan	EUR	lmi	0.72	0.60	0.84	16
St. Vincent & the Grenadines	AMR	umi	0.6	0.48	0.72	16
Venezuela, Bolivarian Republic of	AMR		0.6	0.48	0.72	16
Viet Nam	WPR	lmi	0.6	0.48	0.72	16
Vanuatu	WPR	lmi	0.6	0.48	0.72	16
Samoa	WPR		0.6	0.48	0.72	16
Kosovo	EUR	umi	0.72	0.60	0.84	16
Yemen	EMR	low	0.6	0.48	0.72	16 (SEAR average) *
South Africa	AFR	umi	0.53	0.41	0.65	16
Zambia	AFR	lmi	0.53	0.41	0.65	16
Zimbabwe	AFR	lmi	0.53	0.41	0.65	16

Table S5: Type distribution assumptions for vaginal cancer.

Note: Asterisks (*) denote instances where the authors performed calculations to determine the average. AFR= African Region; AMR= Region of the Americas; EMR= Eastern Mediterranean Region; EUR= European Region; lb= lower bound; lmi= lower-middle income; ub= upper bound; umi= upper-middle income; WPR= Western Pacific Region.

Country Name	Region	Income Level	Type distribution mean	Lower Bound	Upper Bound	Source
Afghanistan	EMR	low	0.76	0.61	0.91	16 (SEAR average) *
Angola	AFR	lmi	0.92	0.77	1.00	16
Albania	EUR	umi	0.82	0.67	0.97	16
Armenia	EUR	umi	0.82	0.67	0.97	16
Azerbaijan	EUR	umi	0.82	0.67	0.97	16
Burundi	AFR	low	0.92	0.77	1.00	16
Benin	AFR	lmi	0.92	0.77	1.00	16
Burkina Faso	AFR	low	0.92	0.77	1.00	16
Bangladesh	SEAR	lmi	0.76	0.61	0.91	16
Bosnia and Herzegovina	EUR	umi	0.82	0.67	0.97	16
Belarus	EUR	umi	0.82	0.67	0.97	16
Belize	AMR	umi	0.71	0.56	0.86	16
Bolivia, Plurinational State of	AMR	lmi	0.71	0.56	0.86	16
Bhutan	SEAR	lmi	0.76	0.61	0.91	16
Central African Republic	AFR	low	0.92	0.77	1.00	16
China (Asia)	WPR	umi	0.76	0.61	0.91	16
Cote d'Ivoire	AFR	lmi	0.92	0.77	1.00	16
Cameroon	AFR	lmi	0.92	0.77	1.00	16
Congo, the Democratic Republic of the	AFR	low	0.92	0.77	1.00	16
Congo	AFR	lmi	0.92	0.77	1.00	16
Colombia	AMR	umi	0.71	0.56	0.86	16
Comoros	AFR	lmi	0.92	0.77	1.00	16
Cabo Verde	AFR	lmi	0.92	0.77	1.00	16
Cuba	AMR	umi	0.71	0.56	0.86	16
Djibouti	EMR	lmi	0.92	0.77	1.00	16 (AFR average) *
Dominica	AMR	umi	0.71	0.56	0.86	16
Algeria	AFR	lmi	0.92	0.77	1.00	16
Ecuador	AMR	umi	0.71	0.56	0.86	16
Egypt	EMR	lmi	0.92	0.77	1.00	16 (AFR average) *
Eritrea	AFR	low	0.92	0.77	1.00	16
Ethiopia	AFR	low	0.92	0.77	1.00	16
Fiji	WPR	umi	0.76	0.61	0.91	16

Micronesia, Federated States of	WPR	lmi	0.76	0.61	0.91	16
Georgia	EUR	umi	0.82	0.67	0.97	16
Ghana	AFR	lmi	0.92	0.77	1.00	16
Guinea	AFR	lmi	0.92	0.77	1.00	16
Gambia	AFR	low	0.92	0.77	1.00	16
Guinea-Bissau	AFR	low	0.92	0.77	1.00	16
Grenada	AMR	umi	0.71	0.56	0.86	16
Guatemala	AMR	umi	0.71	0.56	0.86	16
Guyana	AMR		0.71	0.56	0.86	16
Honduras	AMR	lmi	0.71	0.56	0.86	16
Haiti	AMR	lmi	0.71	0.56	0.86	16
Indonesia	SEAR	umi	0.76	0.61	0.91	16
India	SEAR	lmi	0.76	0.61	0.91	16
Iran, Islamic Republic of	EMR	lmi	0.92	0.77	1.00	16 (SEAR average) *
Iraq	EMR	umi	0.76	0.61	0.91	16 (SEAR average) *
Jamaica	AMR	umi	0.71	0.56	0.86	16
Jordan	EMR	lmi	0.76	0.61	0.91	16
Kenya	AFR	lmi	0.92	0.77	1.00	16
Kyrgyzstan	EUR		0.82	0.67	0.97	16
Cambodia	WPR	lmi	0.76	0.61	0.91	16
Kiribati	WPR	lmi	0.76	0.61	0.91	16
Lao People's Democratic Republic	WPR	lmi	0.76	0.61	0.91	16
Liberia	AFR	low	0.92	0.77	1.00	16
St. Lucia	AMR	umi	0.71	0.56	0.86	16
Sri Lanka	SEAR	lmi	0.76	0.61	0.91	16
Lesotho	AFR	lmi	0.92	0.77	1.00	16
Morocco	EMR	lmi	0.92	0.77	1.00	16 (AFR average) *
Moldova, Republic of	EUR	umi	0.82	0.67	0.97	16
Madagascar	AFR	low	0.92	0.77	1.00	16
Maldives	SEAR	umi	0.76	0.61	0.91	16
Marshall Islands	WPR	umi	0.76	0.61	0.91	16
Macedonia, the former Yugoslav Republic of	EUR		0.82	0.67	0.97	16
Mali	AFR	low	0.92	0.77	1.00	16

Myanmar	SEAR	lmi	0.76	0.61	0.91	16
Mongolia	WPR	lmi	0.76	0.61	0.91	16
Mozambique	AFR	low	0.92	0.77	1.00	16
Mauritania	AFR	lmi	0.92	0.77	1.00	16
Malawi	AFR	low	0.92	0.77	1.00	16
Namibia	AFR	umi	0.92	0.77	1.00	16
Niger	AFR	low	0.92	0.77	1.00	16
Nigeria	AFR	lmi	0.92	0.77	1.00	16
Nicaragua	AMR	lmi	0.71	0.56	0.86	16
Nepal	SEAR	lmi	0.76	0.61	0.91	16
Pakistan	EMR	lmi	0.76	0.61	0.91	16 (SEAR average) *
Peru	AMR	umi	0.71	0.56	0.86	16
Philippines (Asia)	WPR	lmi	0.76	0.61	0.91	16
Papua New Guinea	WPR	lmi	0.76	0.61	0.91	16
Korea, Democratic People's Republic of	SEAR	low	0.76	0.61	0.91	16
Paraguay	AMR	umi	0.71	0.56	0.86	16
Palestine, State of	EMR	lmi	0.76	0.61	0.91	16 (SEAR average) *
Rwanda	AFR	low	0.92	0.77	1.00	16
Sudan	EMR	low	0.92	0.77	1.00	16 (AFR average) *
Senegal	AFR	lmi	0.92	0.77	1.00	16
Solomon Islands	WPR	lmi	0.76	0.61	0.91	16
Sierra Leone	AFR	low	0.92	0.77	1.00	16
El Salvador	AMR	umi	0.71	0.56	0.86	16
Somalia	EMR	low	0.92	0.77	1.00	16 (AFR average) *
Serbia	EUR	umi	0.82	0.67	0.97	16
South Sudan	AFR	low	0.92	0.77	1.00	16
Sao Tome and Principe	AFR	lmi	0.92	0.77	1.00	16
Swaziland	AFR		0.92	0.77	1.00	16
Syrian Arab Republic	EMR	low	0.76	0.61	0.91	16 (SEAR average) *
Chad	AFR	low	0.92	0.77	1.00	16
Togo	AFR	low	0.92	0.77	1.00	16
Thailand	SEAR	umi	0.76	0.61	0.91	16 (SEAR average) *
Tajikistan	EUR	lmi	0.82	0.67	0.97	16
Turkmenistan	EUR	umi	0.82	0.67	0.97	16
Timor-Leste	SEAR	lmi	0.76	0.61	0.91	16

Tonga	WPR	umi	0.76	0.61	0.91	16
Tunisia	EMR	lmi	0.92	0.77	1.00	16 (AFR average) *
Tuvalu	WPR	umi	0.76	0.61	0.91	16
Tanzania, United Republic of	AFR	lmi	0.92	0.77	1.00	16
Uganda	AFR	low	0.92	0.77	1.00	16
Ukraine	EUR	lmi	0.82	0.67	0.97	16
Uzbekistan	EUR	lmi	0.82	0.67	0.97	16
St. Vincent & the Grenadines	AMR	umi	0.71	0.56	0.86	16
Venezuela, Bolivarian Republic of	AMR		0.71	0.56	0.86	16
Viet Nam	WPR	lmi	0.76	0.61	0.91	16
Vanuatu	WPR	lmi	0.76	0.61	0.91	16
Samoa	WPR		0.76	0.61	0.91	16
Kosovo	EUR	umi	0.82	0.67	0.97	16
Yemen	EMR	low	0.76	0.61	0.91	16 (SEAR average) *
South Africa	AFR	umi	0.92	0.77	1.00	16
Zambia	AFR	lmi	0.92	0.77	1.00	16
Zimbabwe	AFR	lmi	0.92	0.77	1.00	16

Table S6: Type distribution assumptions for vulvar cancer.

Note: Asterisks (*) denote instances where the authors performed calculations to determine the average. AFR= African Region; AMR= Region of the Americas; EMR= Eastern Mediterranean Region; EUR= European Region; lb= lower bound; lmi= lower-middle income; ub= upper bound; umi= upper-middle income; WPR= Western Pacific Region.

Country Name	Region	Income Level	Probability of Death for Anal Cancer (lb, ub)	Probability of Death for Oropharyngeal Cancer (lb, ub)	Probability of Death for Vaginal Cancer (lb, ub)	Probability of Death for Vulvar Cancer (lb, ub)	Source
Afghanistan	EMR	low	0.88 (0.79, 0.97) *	0.87 (0.81, 0.93)	0.92 (0.87, 0.97)	0.88 (0.75, 1)	19
Angola	AFR	lmi	0.76 (0.64, 0.87)	0.93 (0.84, 1)	0.74 (0.65, 0.83)	0.55 (0.36, 0.74)	19
Albania	EUR	umi	1 (0.85, 1)	0.57 (0.45, 0.68)	1 (0.88, 1) *	0.43 (0.17, 0.68)	19
Armenia	EUR	umi	1 (0.85, 1) *	1 (0.88, 1) *	0.94 (0.82, 1)	0.64 (0.38, 0.89)	19
Azerbaijan	EUR	umi	0.65 (0.5, 0.8)	0.77 (0.65, 0.89)	0.96 (0.84, 1)	0.85 (0.6, 1)	19
Burundi	AFR	low	0.7 (0.59, 0.82)	0.95 (0.86, 1)	0.88 (0.79, 0.96)	0.59 (0.4, 0.79)	19
Benin	AFR	lmi	0.88 (0.77, 0.99)	0.82 (0.73, 0.91)	0.89 (0.8, 0.97)	0.76 (0.56, 0.95)	19
Burkina Faso	AFR	low	0.86 (0.75, 0.97)	0.91 (0.82, 1)	0.77 (0.68, 0.86)	0.7 (0.51, 0.89)	19
Bangladesh	SEAR	lmi	0.93 (0.85, 1)	0.64 (0.52, 0.75)	0.86 (0.76, 0.96)	0.84 (0.74, 0.95)	19
Bosnia and Herzegovina	EUR	umi	0.84 (0.68, 0.99)	0.79 (0.67, 0.9)	0.7 (0.58, 0.81)	0.24 (0, 0.49)	19
Belarus	EUR	umi	0.55 (0.4, 0.71)	0.71 (0.6, 0.83)	0.84 (0.72, 0.95)	0.21 (0, 0.46)	19
Belize	AMR	umi	1 (0.87, 1) *	1 (0.88, 1) *	1 (0.91, 1) *	1 (0.77, 1)*	19
Bolivia, Plurinational State of	AMR	lmi	1 (0.87, 1) *	0.81 (0.69, 0.93)	0.83 (0.74, 0.91)	0.86 (0.63, 1)	19
Bhutan	SEAR	lmi	1 (0.92, 1) *	0.76 (0.64, 0.87)	0.76 (0.66, 0.86)	1 (0.89, 1) *	19
Central African Republic	AFR	low	0.75 (0.64, 0.86)	0.96 (0.87, 1)	0.89 (0.8, 0.97)	0.69 (0.5, 0.88)	19
China (Asia)	WPR	umi	0.86 (0.75, 0.97)	0.93 (0.86, 1)	0.9 (0.76, 1)	0.84 (0.66, 1)	19
Cote d'Ivoire	AFR	lmi	0.76 (0.64, 0.87)	0.92 (0.83, 1.01)	0.8 (0.72, 0.89)	0.87 (0.68, 1)	19
Cameroon	AFR	lmi	0.77 (0.66, 0.11)	0.87 (0.78, 0.96)	0.81 (0.72, 0.9)	0.79 (0.6, 0.99)	19
Congo, the Democratic Republic of the	AFR	low	0.76 (0.64, 0.87)	0.95 (0.86, 1)	0.82 (0.73, 0.91)	0.66 (0.46, 0.85)	19

Congo	AFR	lmi	0.65 (0.54, 0.76)	1 (0.91, 1) *	0.9 (0.81, 0.98)	0.7 (0.51, 0.89)	19 (assumption for survival rate of all cancers based on AFR)
Colombia	AMR	umi	0.78 (0.65, 0.91)	0.79 (0.68, 0.91)	0.84 (0.75, 0.92)	0.59 (0.36, 0.83)	19
Comoros	AFR	lmi	0.79 (0.68, 0.91)	1 (0.91, 1) *	1 (0.91, 1) *	0.39 (0.2, 0.59)	19
Cabo Verde	AFR	lmi	0.79 (0.68, 0.91)	0.49 (0.4, 0.58)	1 (0.91, 1) *	0.7 (0.51, 0.89)	19
Cuba	AMR	umi	0.56 (0.43, 0.69)	0.58 (0.47, 0.7)	0.67 (0.58, 0.76)	0.5 (0.26, 0.73)	19
Djibouti	EMR	lmi	1 (0.91, 1) *	1 (0.94, 1) *	1 (0.95, 1) *	0.81 (0.68, 0.94)	19
Dominica	AMR	umi	0.82 (0.69, 0.95)	0.86 (0.74, 0.98)	0.84 (0.76, 0.93)	0.7 (0.46, 0.93)	19 (assumption for survival rate of anal, and oropharyngeal cancers based on AMR)
Algeria	AFR	lmi	0.9 (0.79, 1)	0.96 (0.87, 1)	0.92 (0.84, 1)	0.86 (0.67, 1)	19
Ecuador	AMR	umi	0.8 (0.68, 0.93)	0.87 (0.75, 0.99)	0.9 (0.81, 0.98)	0.76 (0.52, 0.99)	19
Egypt	EMR	lmi	0.91 (0.82, 1)	0.93 (0.88, 0.99)	0.93 (0.88, 0.98)	0.8 (0.67, 0.93)	19
Eritrea	AFR	low	0.9 (0.78, 1.01)	1 (0.91, 1) *	0.84 (0.76, 0.93)	0.6 (0.41, 0.79)	19
Ethiopia	AFR	low	0.81 (0.7, 0.92)	0.97 (0.88, 1)	0.79 (0.7, 0.87)	0.67 (0.48, 0.86)	19

Fiji	WPR	umi	1 (0.89, 1) *	1 (0.93, 1) *	0.79 (0.65, 0.94)	1 (0.81, 1) *	19
Micronesia, Federated States of	WPR	lmi	0.85 (0.74, 0.96)	0.88 (0.81, 0.95)	0.78 (0.63, 0.92)	0.34 (0.15, 0.53)	19 (assumption for survival rate of anal cancer based on WPR)
Georgia	EUR	umi	0.9 (0.75, 1.06)	0.9 (0.79, 1)	0.68 (0.57, 0.8)	0.28 (0.02, 0.53)	19
Ghana	AFR	lmi	0.64 (0.53, 0.76)	0.9 (0.81, 1)	0.93 (0.85, 1)	0.82 (0.63, 1)	19
Guinea	AFR	lmi	0.73 (0.62, 0.85)	0.9 (0.81, 1)	0.86 (0.77, 0.95)	0.63 (0.43, 0.82)	19 (assumption for survival rate of all cancers based on AFR)
Gambia	AFR	low	0.92 (0.81, 1)	1 (0.91, 1) *	1 (0.91, 1) *	1 (0.81, 1) *	19
Guinea-Bissau	AFR	low	0.9 (0.79, 1.02)	1 (0.91, 1) *	0.82 (0.73, 0.91)	0.82 (0.63, 1)	19
Grenada	AMR	umi	0.82 (0.69, 0.95)	0.86 (0.74, 0.98)	0.85 (0.77, 0.94)	0.71 (0.48, 0.95)	19 (assumption for survival rate of anal and oropharyngeal cancers based on AMR)
Guatemala	AMR	umi	0.99 (0.86, 1)	0.88 (0.76, 1)	0.93 (0.85, 1)	0.93 (0.7, 1)	19
Guyana	AMR		1 (0.87, 1) *	1 (0.88, 1) *	1 (0.91, 1) *	1 (0.77, 1) *	19

Honduras	AMR	lmi	0.74 (0.61, 0.87)	0.85 (0.73, 0.97)	0.85 (0.77, 0.94)	0.89 (0.65, 1)	19
Haiti	AMR	lmi	0.98 (0.85, 1)	0.65 (0.53, 0.77)	0.83 (0.74, 0.91)	0.97 (0.74, 1)	19
Indonesia	SEAR	umi	0.92 (0.84, 1)	0.89 (0.77, 1)	0.85 (0.75, 0.95)	0.72 (0.61, 0.82)	19
India	SEAR	lmi	0.84 (0.75, 0.92)	0.73 (0.62, 0.85)	0.66 (0.56, 0.76)	0.77 (0.67, 0.88)	19
Iran, Islamic Republic of	EMR	lmi	0.93 (0.84, 1)	0.91 (0.86, 0.97)	0.97 (0.92, 1)	0.97 (0.84, 1)	19
Iraq	EMR	umi	0.95 (0.86, 1)	0.98 (0.92, 1)	0.95 (0.9, 1)	0.93 (0.8, 1)	19
Jamaica	AMR	umi	0.82 (0.69, 0.95)	1 (0.88, 1) *	0.88 (0.79, 0.96)	0.82 (0.59, 1)	19
Jordan	EMR	lmi	0.94 (0.85, 1)	0.98 (0.92, 1)	0.87 (0.82, 0.92)	0.9 (0.78, 1)	19
Kenya	AFR	lmi	0.69 (0.58, 0.8)	0.96 (0.87, 1)	0.88 (0.79, 0.96)	0.72 (0.53, 0.91)	19
Kyrgyzstan	EUR		0.86 (0.71, 1)	0.89 (0.77, 1)	0.91 (0.8, 1)	0.64 (0.38, 0.89)	19
Cambodia	WPR	lmi	0.91 (0.8, 1)	0.85 (0.78, 0.92)	0.93 (0.79, 1)	0.84 (0.65, 1)	19
Kiribati	WPR	lmi	0.85 (0.74, 0.96)	0.88 (0.81, 0.95)	0.8 (0.66, 0.95)	0.34 (0.15, 0.53)	19 (assumption for survival rate for vaginal and vulvar cancer based on WPR)
Lao People's Democratic Republic	WPR	lmi	0.9 (0.78, 1)	0.9 (0.83, 0.96)	0.84 (0.7, 0.99)	0.78 (0.59, 0.97)	19
Liberia	AFR	low	0.82 (0.71, 0.93)	0.96 (0.87, 1)	0.76 (0.67, 0.84)	0.89 (0.69, 1)	19
St. Lucia	AMR	umi	1 (0.87, 1) *	1 (0.88, 1) *	1 (0.91, 1) *	1 (0.77, 1) *	19
Sri Lanka	SEAR	lmi	0.81 (0.73, 0.89)	0.85 (0.74, 0.97)	0.79 (0.69, 0.89)	0.82 (0.71, 0.92)	19
Lesotho	AFR	lmi	0.84 (0.72, 0.95)	1 (0.91, 1) *	0.84 (0.75, 0.92)	0.21 (0.02, 0.4)	19

Morocco	EMR	lmi	0.69 (0.6, 0.78)	0.9 (0.85, 0.96)	0.81 (0.76, 0.86)	0.56 (0.43, 0.69)	19
Moldova, Republic of	EUR	umi	0.87 (0.72, 1)	0.79 (0.67, 0.9)	0.87 (0.75, 0.99)	0.29 (0.03, 0.54)	19
Madagascar	AFR	low	0.51 (0.4, 0.62)	0.94 (0.85, 1)	0.8 (0.72, 0.89)	0.44 (0.25, 0.64)	19
Maldives	SEAR	umi	1 (0.92, 1) *	1 (0.89, 1) *	1 (0.9, 1) *	1 (0.89, 1) *	19
Marshall Islands	WPR	umi	0.93 (0.82, 1)	1 (0.93, 1) *	1 (0.85, 1) *	1 (0.81, 1) *	19
Macedonia, the former Yugoslav Republic of	EUR		1 (0.85, 1) *	0.9 (0.79, 1)	0.83 (0.71, 0.94)	0.51 (0.26, 0.77)	19
Mali	AFR	low	0.8 (0.69, 0.92)	0.89 (0.8, 0.98)	0.82 (0.73, 0.91)	0.77 (0.58, 0.96)	19
Myanmar	SEAR	lmi	0.9 (0.81, 0.98)	0.87 (0.76, 0.98)	0.86 (0.76, 0.96)	0.83 (0.72, 0.93)	19
Mongolia	WPR	lmi	0.89 (0.78, 1)	0.79 (0.72, 0.85)	0.74 (0.59, 0.89)	0.79 (0.6, 0.97)	19
Mozambique	AFR	low	0.61 (0.5, 0.73)	0.98 (0.89, 1)	0.84 (0.76, 0.93)	0.42 (0.23, 0.61)	19
Mauritania	AFR	lmi	0.7 (0.59, 0.82)	0.96 (0.87, 1)	0.8 (0.72, 0.89)	0.7 (0.51, 0.9)	19
Malawi	AFR	low	0.53 (0.42, 0.65)	1 (0.91, 1) *	0.58 (0.5, 0.67)	0.54 (0.35, 0.73)	19
Namibia	AFR	umi	0.79 (0.68, 0.91)	0.73 (0.64, 0.82)	0.54 (0.46, 0.63)	0.4 (0.21, 0.59)	19
Niger	AFR	low	0.76 (0.64, 0.87)	0.95 (0.86, 1)	0.82 (0.73, 0.91)	0.66 (0.46, 0.85)	19 (assumption for survival rate of all cancers based on AFR)
Nigeria	AFR	lmi	0.76 (0.65, 0.88)	0.93 (0.84, 1)	0.91 (0.83, 1)	0.59 (0.4, 0.79)	19
Nicaragua	AMR	lmi	0.81 (0.68, 0.94)	1 (0.88, 1) *	0.81 (0.72, 0.9)	0.79 (0.55, 1)	19
Nepal	SEAR	lmi	0.94 (0.86, 1)	0.93 (0.82, 1)	0.89 (0.79, 0.99)	0.94 (0.84, 1)	19
Pakistan	EMR	lmi	0.93 (0.84, 1)	0.88 (0.82, 0.94)	0.88 (0.83, 0.93)	0.9 (0.77, 1)	19

Peru	AMR	umi	0.73 (0.61, 0.86)	0.9 (0.79, 1)	0.84 (0.76, 0.93)	0.66 (0.43, 0.9)	19
Philippines (Asia)	WPR	lmi	0.84 (0.73, 0.96)	0.9 (0.84, 0.97)	0.83 (0.68, 0.97)	0.88 (0.69, 1)	19 (assumption for survival rate of all cancers based on WPR)
Papua New Guinea	WPR	lmi	0.62 (0.51, 0.73)	0.91 (0.85, 0.98)	0.71 (0.57, 0.86)	0.87 (0.68, 1)	19
Korea, Democratic People's Republic of	SEAR	low	0.79 (0.71, 0.88)	0.93 (0.82, 1)	0.89 (0.79, 0.99)	0.85 (0.75, 0.96)	19
Paraguay	AMR	umi	0.77 (0.64, 0.9)	0.94 (0.82, 1)	1 (0.91, 1) *	0.71 (0.48, 0.95)	19
Palestine, State of	EMR	lmi	0.89 (0.8, 0.98)	0.93 (0.88, 0.99)	0.89 (0.84, 0.94)	0.79 (0.66, 0.92)	19 (assumption for survival rate of all cancers based on EMR)
Rwanda	AFR	low	0.84 (0.72, 0.95)	0.84 (0.75, 0.93)	0.9 (0.81, 0.98)	0.54 (0.35, 0.74)	19
Sudan	EMR	low	0.9 (0.82, 0.99)	0.97 (0.91, 1)	0.9 (0.81, 0.96)	0.7 (0.57, 0.83)	19
Senegal	AFR	lmi	0.76 (0.64, 0.87)	0.94 (0.85, 1)	0.82 (0.73, 0.91)	0.76 (0.57, 0.96)	19
Solomon Islands	WPR	lmi	1 (0.89, 1) *	1 (0.93, 1) *	1 (0.85, 1) *	1 (0.81, 1) *	19
Sierra Leone	AFR	low	0.79 (0.68, 0.91)	0.95 (0.86, 1)	0.82 (0.73, 0.91)	0.74 (0.55, 0.93)	19
El Salvador	AMR	umi	0.84 (0.72, 0.97)	1 (0.88, 1) *	0.87 (0.78, 0.96)	0.65 (0.42, 0.88)	19
Somalia	EMR	low	0.79 (0.71, 0.88)	0.96 (0.9, 1)	0.81 (0.76, 0.86)	0.68 (0.55, 0.81)	19

Serbia	EUR	umi	0.67 (0.52, 0.82)	0.68 (0.57, 0.8)	0.64 (0.52, 0.75)	0.14 (0, 0.39)	19
South Sudan	AFR	low	0.76 (0.64, 0.87)	0.95 (0.86, 1)	0.82 (0.73, 0.91)	0.66 (0.46, 0.85)	19 (assumption for survival rate of all cancers based on AFR)
Sao Tome and Principe	AFR	lmi	1 (0.89, 1) *	1 (0.91, 1) *	1 (0.91, 1) *	1 (0.81, 1)	19
Swaziland	AFR		0.84 (0.73, 0.96)	1 (0.91, 1) *	0.84 (0.76, 0.93)	0.13 (0, 0.32)	19
Syrian Arab Republic	EMR	low	0.94 (0.85, 1)	0.94 (0.88, 1)	0.9 (0.76, 0.95)	0.74 (0.61, 0.87)	19
Chad	AFR	low	0.83 (0.71, 0.94)	0.94 (0.85, 1)	0.88 (0.79, 0.96)	0.76 (0.57, 0.96)	19
Togo	AFR	low	0.76 (0.64, 0.87)	0.95 (0.86, 1)	0.82 (0.73, 0.91)	0.66 (0.47, 0.86)	19 (assumption for survival rate of anal, oropharyngeal, and vaginal cancers based on AFR)
Thailand	SEAR	umi	0.84 (0.75, 0.92)	0.8 (0.69, 0.92)	0.79 (0.7, 0.89)	0.78 (0.67, 0.88)	19
Tajikistan	EUR	lmi	0.9 (0.75, 1)	0.84 (0.73, 0.96)	1 (0.88, 1) *	0.92 (0.67, 1)	19
Turkmenistan	EUR	umi	0.72 (0.57, 0.87)	0.63 (0.51, 0.75)	0.88 (0.76, 1)	0.72 (0.46, 0.97)	19
Timor-Leste	SEAR	lmi	1 (0.92, 1) *	1 (0.89, 1) *	1 (0.9, 1) *	1 (0.89, 1) *	19
Tonga	WPR	umi	0.93 (0.82, 1)	0.98 (0.91, 1)	0.85 (0.71, 1)	0.84 (0.66, 1)	19

Tunisia	EMR	lmi	0.75 (0.66, 0.84)	0.97 (0.91, 1)	0.9 (0.85, 0.96)	0.73 (0.6, 0.86)	19
Tuvalu	WPR	umi	0.93 (0.82, 1)	0.98 (0.91, 1)	0.85 (0.71, 1)	0.84 (0.66, 1)	19
Tanzania, United Republic of	AFR	lmi	0.46 (0.35, 0.57)	0.94 (0.85, 1)	0.77 (0.68, 0.86)	0.55 (0.36, 0.75)	19
Uganda	AFR	low	0.88 (0.77, 0.99)	0.96 (0.87, 1)	0.9 (0.82, 0.99)	0.73 (0.54, 0.93)	19
Ukraine	EUR	lmi	0.66 (0.51, 0.81)	0.7 (0.59, 0.82)	0.7 (0.59, 0.82)	0.22 (0, 0.48)	19
Uzbekistan	EUR	lmi	0.65 (0.5, 0.8)	0.7 (0.58, 0.81)	0.9 (0.79, 1)	0.81 (0.56, 1)	19
St. Vincent & the Grenadines	AMR	umi	0.82 (0.69, 0.95)	0.87 (0.75, 0.99)	0.85 (0.77, 0.94)	0.71 (0.48, 0.95)	19 (assumption for survival rate of anal, vaginal, and vulvar cancers based on AMR)
Venezuela, Bolivarian Republic of	AMR		0.66 (0.53, 0.78)	0.83 (0.71, 0.94)	0.79 (0.7, 0.87)	0.76 (0.52, 0.99)	19
Viet Nam	WPR	lmi	0.71 (0.6, 0.82)	0.9 (0.84, 0.97)	0.94 (0.8, 1)	0.87 (0.68, 1)	19
Vanuatu	WPR	lmi	1 (0.89, 1) *	1 (0.93, 1) *	0.52 (0.37, 0.66)	1 (0.81, 1) *	19
Samoa	WPR		1 (0.89, 1) *	1 (0.93, 1) *	1 (0.85, 1) *	1 (0.81, 1) *	19
Kosovo	EUR	umi	0.76 (0.61, 0.91)	0.76 (0.64, 0.87)	0.82 (0.7, 0.94)	0.41 (0.15, 0.66)	19 (assumption for survival rate of all cancers based on EUR)

Yemen	EMR	low	0.95 (0.86, 1)	0.79 (0.74, 0.85)	0.85 (0.74, 0.97)	0.93 (0.8, 1)	19
South Africa	AFR	umi	0.8 (0.69, 0.92)	0.84 (0.75, 0.93)	0.83 (0.74, 0.91)	0.58 (0.38, 0.77)	19
Zambia	AFR	lmi	0.71 (0.6, 0.82)	0.99 (0.9, 1)	0.77 (0.68, 0.86)	0.44 (0.25, 0.63)	19
Zimbabwe	AFR	lmi	0.56 (0.45, 0.67)	0.91 (0.82, 1)	0.76 (0.67, 0.84)	0.38 (0.19, 0.57)	19

Table S7: Probability of death and uncertainty bounds across countries in analysis.

Note: * Indicates where adjustments were made for the survival rate bounds. Calculations for probability of death and its bounds were based on the relative mortality rates (per 100,000 women per year) from the HPV Stats database.[19] AFR= African Region; AMR= Region of the Americas; EMR= Eastern Mediterranean Region; EUR= European Region; lb= lower bound; lmi= lower-middle income; ub= upper bound; umi= upper-middle income; WPR= Western Pacific Region.

CUMULATIVE CASES AVERTED BY COUNTRY ACROSS 2030–2100

COUNTRY	ANAL	OROPHARYNGEAL	VAGINAL	VULVAR
AFGHANISTAN	6,860 (5,450–8,380)	6,560 (4,010–8,600)	1,970 (1,430–2,600)	5,180 (3,850–6,610)
ANGOLA	2,730 (1,880–3,770)	9,580 (5,150–14,200)	8,140 (5,410–11,500)	11,100 (7,290–15,500)
ALBANIA	42 (25–63)	7 (2–13)	77 (53–105)	225 (167–283)
ARMENIA	88 (54–127)	NA	92 (57–134)	243 (145–354)
AZERBAIJAN	928 (570–1,320)	48 (11–87)	33 (16–51)	549 (353–754)
BURUNDI	3,930 (2,830–4,900)	135 (36–262)	1,480 (1,050–1,930)	8,570 (6,010–10,900)
BENIN	1,910 (1,490–2,350)	2,130 (1,090–2,940)	1,630 (1,210–2,040)	3,830 (3,010–4,830)
BURKINA FASO	13,400 (9,010–18,000)	NA	2,890 (782–5,090)	4,670 (2,700–6,720)
BANGLADESH	4,940 (3,870–6,100)	4,240 (1,350–7,700)	6,620 (4,900–8,560)	13,700 (10,700–17,100)
BOSNIA AND HERZEGOVINA	57 (35–81)	101 (47–152)	95 (66–123)	248 (164–335)
BELARUS	931 (674–1,170)	69 (22–130)	183 (132–240)	1,690 (1,240–2,140)
BELIZE	NA	NA	NA	NA
BOLIVIA, PLURINATIONAL STATE OF	401 (244–577)	1,810 (572–3,570)	1,300 (692–1,900)	1,470 (840–2,090)
BHUTAN	NA	313 (178–490)	NA	NA
CENTRAL AFRICAN REPUBLIC	1,420 (1,040–1,810)	NA	393 (105–692)	2,610 (1,890–3,370)
CHINA	21,600 (17,100–26,800)	11,900 (5,570–17,800)	18,600 (14,200–23,200)	31,400 (24,100–39,500)
COTE D'IVOIRE	7,280 (5,270–9,240)	2,130 (1,050–3,330)	5,970 (3,990–8,040)	4,090 (2,790–5,390)
CAMEROON	5,570 (4,170–7,030)	483 (149–851)	3,400 (2,290–4,460)	6,410 (4,840–7,930)
CONGO, THE DEMOCRATIC REPUBLIC OF THE	42,300 (32,700–52,300)	443 (126–799)	12,800 (8,980–16,500)	63,900 (48,800–77,100)
CONGO	1,750 (1,250–2,240)	197 (62–349)	420 (291–581)	2,220 (1,650–2,950)
COLOMBIA	10,900 (10,300–11,600)	707 (202–1,190)	3,520 (2,920–4,130)	11,900 (10,800–13,000)
COMOROS	NA	NA	NA	507 (396–619)
CABO VERDE (CAPE VERDE)	43 (11–73)	NA	NA	35 (10–61)
CUBA	1,450 (1,020–1,850)	89 (29–162)	412 (288–534)	1,280 (987–1,550)
DJIBOUTI	NA	NA	51 (25–78)	190 (125–258)
DOMINICA	3 (0–5)	1 (0–3)	8 (4–13)	2 (1–3)
ALGERIA	2,890 (2,190–3,590)	690 (403–960)	1,630 (1,250–2,030)	3,470 (2,540–4,570)
ECUADOR	4,690 (3,310–6,200)	256 (88–447)	1,000 (708–1,320)	4,310 (3,340–5,580)
EGYPT	6,500 (5,010–7,850)	859 (331–1,520)	3,840 (2,710–5,110)	15,600 (11,800–20,400)
ERITREA	325 (196–464)	NA	400 (239–576)	1,310 (939–1,710)

CUMULATIVE CASES AVERTED BY COUNTRY ACROSS 2030–2100

COUNTRY	ANAL	OROPHARYNGEAL	VAGINAL	VULVAR
ETHIOPIA	27,700 (19,600–35,000)	4,650 (2,730–6,780)	25,900 (18,500–33,500)	61,300 (48,700–75,700)
FIJI	247 (147–343)	8 (1–16)	66 (30–98)	NA
MICRONESIA, FEDERATED STATES OF	NA	NA	NA	NA
GEORGIA	71 (42–98)	11 (4–22)	113 (77–155)	773 (592–971)
GHANA	10,700 (7,040–14,500)	289 (70–652)	909 (478–1,450)	6,480 (4,970–8,340)
GUINEA	1,250 (917–1,580)	273 (115–449)	1,570 (1,080–2,010)	1,100 (850–1,410)
GAMBIA	NA	NA	NA	139 (39–234)
GUINEA-BISSAU	NA	NA	80 (21–136)	68 (22–115)
GRENADA	19 (15–23)	3 (1–6)	6 (3–8)	16 (11–20)
GUATEMALA	395 (215–555)	1,120 (545–1,670)	728 (469–967)	822 (531–1,120)
GUYANA	24 (7–44)	NA	NA	72 (41–107)
HONDURAS	2,190 (1,520–2,850)	419 (241–599)	1,020 (628–1,360)	1,610 (1,160–2,050)
HAITI	450 (211–680)	1,600 (946–2,190)	827 (549–1,130)	133 (63–208)
INDONESIA	10,100 (7,570–13,300)	13,500 (8,770–17,800)	9,080 (6,710–12,100)	38,400 (29,000–49,700)
INDIA	95,300 (72,100–118,000)	143,000 (92,600–189,000)	133,000 (98,500–177,000)	133,000 (103,000–169,000)
IRAN, ISLAMIC REPUBLIC OF	3,100 (2,730–3,450)	1,570 (715–2,200)	1,250 (1,020–1,510)	1,280 (1,020–1,560)
IRAQ	2,180 (1,670–2,760)	516 (242–787)	1,540 (1,060–2,030)	3,060 (2,420–3,870)
JAMAICA	119 (90–147)	6 (1–13)	53 (37–74)	102 (70–127)
JORDAN	758 (532–988)	225 (120–350)	725 (559–939)	892 (662–1,120)
KENYA	19,000 (13,900–24,900)	1,900 (1,180–2,660)	3,990 (2,890–5,420)	13,600 (10,100–17,600)
KYRGYZSTAN	507 (368–643)	227 (105–398)	345 (160–517)	1,490 (1,010–1,970)
CAMBODIA	757 (498–998)	196 (82–347)	295 (218–389)	2,050 (1,320–2,770)
KIRIBATI	7 (5–9)	16 (9–22)	3 (1–6)	17 (10–24)
LAO PEOPLE'S DEMOCRATIC REPUBLIC	555 (377–700)	549 (320–782)	371 (258–506)	1,160 (877–1,430)
LIBERIA	517 (375–651)	16 (3–35)	597 (426–791)	404 (252–568)
ST LUCIA	NA	NA	NA	NA
SRI LANKA	3,490 (2,630–4,490)	181 (53–323)	741 (517–970)	3,190 (2,110–4,100)
LESOTHO	315 (208–430)	NA	136 (75–206)	1,910 (1,320–2,530)
MOROCCO	3,820 (2,710–4,760)	163 (38–280)	1,440 (1,070–1,840)	7,070 (5,340–8,880)
MOLDOVA, REPUBLIC OF	124 (90–156)	14 (4–26)	24 (14–34)	601 (411–818)
MADAGASCAR	14,100 (11,400–17,000)	258 (74–500)	4,010 (2,970–5,150)	26,400 (19,800–32,300)
MALDIVES	NA	NA	NA	NA

CUMULATIVE CASES AVERTED BY COUNTRY ACROSS 2030–2100

COUNTRY	ANAL	OROPHARYNGEAL	VAGINAL	VULVAR
MARSHALL ISLANDS	NA	NA	NA	3 (0–5)
MACEDONIA (NORTH), THE FORMER YUGOSLAV REPUBLIC OF	134 (94–172)	33 (16–54)	56 (26–86)	128 (77–181)
MALI	6,400 (4,930–7,670)	3,180 (1,790–4,550)	5,800 (4,500–7,220)	7,900 (6,160–9,960)
MYANMAR	1,970 (1,060–2,830)	310 (107–521)	1,140 (662–1,740)	4,280 (3,100–5,620)
MONGOLIA	116 (53–173)	34 (10–61)	329 (234–426)	359 (260–493)
MOZAMBIQUE	13,000 (9,760–16,700)	145 (30–346)	4,370 (2,340–6,480)	40,700 (31,300–52,600)
MAURITANIA	1,310 (1,020–1,620)	259 (123–431)	718 (532–926)	12,800 (7,520–18,100)
MALAWI	10,200 (7,790–12,500)	NA	7,890 (5,940–10,100)	10,200 (7,480–13,100)
NAMIBIA	455 (327–583)	72 (18–134)	606 (423–796)	1,580 (1,190–2,040)
NIGER	7,780 (5,280–10,400)	1,540 (742–2,530)	5,430 (4,030–7,150)	3,430 (2,410–4,470)
NIGERIA	46,800 (34,500–61,300)	10,400 (3,710–19,100)	15,500 (9,930–21,900)	100,000 (74,200–131,000)
NICARAGUA	875 (643–1,150)	397 (232–556)	361 (256–476)	589 (396–784)
NEPAL	786 (520–1,020)	548 (278–803)	1,110 (742–1,500)	946 (635–1,290)
PAKISTAN	19,600 (15,700–24,400)	21,700 (15,300–29,300)	17,100 (12,200–22,800)	21,700 (15,800–28,500)
PERU	1,940 (1,540–2,330)	2,250 (1,270–2,930)	1,590 (1,190–2,000)	2,200 (1,670–2,770)
PHILIPPINES	4,440 (3,330–5,560)	1,070 (343–1,840)	3,340 (2,490–4,460)	8,900 (6,920–11,200)
PAPUA NEW GUINEA	3,380 (2,250–4,460)	400 (92–745)	847 (420–1,260)	985 (623–1,390)
KOREA, DEMOCRATIC PEOPLE'S REPUBLIC OF	391 (292–490)	189 (100–257)	391 (255–530)	889 (652–1,140)
PARAGUAY	2,260 (1,690–2,770)	361 (164–583)	39 (11–65)	1,740 (1,270–2,320)
PALESTINE, STATE OF	526 (402–645)	334 (189–478)	284 (206–377)	973 (686–1,250)
RWANDA	2,310 (1,770–2,870)	51 (18–92)	1,100 (798–1,420)	10,000 (8,440–12,000)
SUDAN	4,700 (2,250–7,170)	1,590 (923–2,370)	4,490 (1,310–7,970)	16,400 (11,300–21,900)
SENEGAL	211 (60–375)	NA	NA	NA
SOLOMON ISLANDS	234 (174–306)	15 (3–24)	130 (92–171)	676 (518–839)
SIERRA LEONE	1,490 (1,140–1,930)	28 (7–52)	937 (700–1,220)	3,020 (2,360–3,720)
EL SALVADOR	395 (293–504)	67 (30–107)	90 (56–127)	279 (201–360)
SOMALIA	4,070 (3,110–5,200)	149 (37–295)	1,390 (905–1,850)	7,270 (5,700–9,040)
SERBIA	818 (302–1,300)	NA	208 (59–365)	NA
SOUTH SUDAN	NA	502 (118–1,170)	1,700 (489–3,000)	4,070 (1,290–7,040)
SAO TOME AND PRINCIPE	22 (15–29)	12 (4–19)	13 (9–18)	53 (38–74)

CUMULATIVE CASES AVERTED BY COUNTRY ACROSS 2030–2100

COUNTRY	ANAL	OROPHARYNGEAL	VAGINAL	VULVAR
SWAZILAND	587 (383–790)	NA	242 (133–364)	1,900 (1,420–2,370)
SYRIAN ARAB REPUBLIC	970 (718–1,290)	418 (228–636)	750 (523–955)	2,790 (1,990–3,730)
CHAD	NA	207 (51–451)	1,980 (836–3,540)	5,830 (2,680–8,720)
TOGO	1,030 (807–1,290)	206 (64–378)	1,340 (912–1,920)	2,320 (1,810–2,940)
THAILAND	1,890 (1,480–2,290)	347 (99–633)	1,310 (962–1,720)	3,500 (2,760–4,310)
TAJIKISTAN	1,340 (990–1,710)	762 (448–1,100)	52 (16–92)	768 (527–1,040)
TURKMENISTAN	1,460 (1,150–1,760)	1,220 (749–1,670)	460 (326–624)	1,170 (881–1,520)
TIMOR-LESTE	NA	NA	NA	NA
TONGA	3 (1–6)	NA	4 (1–7)	7 (4–10)
TUNISIA	1,200 (941–1,460)	164 (84–249)	431 (322–550)	1,470 (1,140–1,800)
TUVALU	NA	NA	NA	NA
TANZANIA, UNITED REPUBLIC OF	57,000 (41,800–70,100)	4,750 (1,330–8,450)	15,900 (11,700–20,400)	46,100 (28,400–61,900)
UGANDA	6,130 (4,520–7,880)	3,560 (1,650–5,470)	5,340 (3,790–7,050)	20,300 (13,900–26,800)
UKRAINE	2,030 (1,460–2,490)	2,000 (1,130–2,720)	946 (692–1,210)	4,680 (3,440–6,050)
UZBEKISTAN	8,560 (6,590–10,600)	4,840 (2,930–6,410)	1,760 (1,250–2,420)	4,380 (3,100–5,650)
ST VINCENT & THE GRENADINES	NA	NA	NA	NA
VENEZUELA, BOLIVARIAN REPUBLIC OF	5,910 (4,260–7,670)	1,780 (992–2,490)	2,540 (1,710–3,370)	2,070 (1,510–2,680)
VIET NAM	7,550 (5,880–9,290)	2,820 (1,860–3,720)	1,030 (774–1,370)	4,000 (3,050–5,360)
VANUATU	110 (74–147)	NA	NA	NA
SAMOA	26 (20–32)	1 (0–3)	18 (11–25)	37 (29–44)
KOSOVO	139 (108–172)	10 (3–16)	61 (44–82)	400 (303–505)
YEMEN	745 (338–1,170)	781 (276–1,540)	3,150 (2,160–4,150)	2,950 (1,960–3,860)
SOUTH AFRICA	3,760 (2,580–4,940)	2,490 (1,410–3,460)	3,090 (2,220–3,970)	2,570 (1,740–3,520)
ZAMBIA	7,840 (5,660–9,870)	122 (40–218)	5,690 (3,690–7,980)	20,100 (15,100–25,300)
ZIMBABWE	5,610 (4,480–7,070)	146 (42–273)	3,160 (2,240–4,250)	17,900 (13,900–22,400)

CUMULATIVE DEATHS AVERTED BY COUNTRY ACROSS 2030–2100

COUNTRY	ANAL	OROPHARYNGEAL	VAGINAL	VULVAR
AFGHANISTAN	5,120 (3,930–6,440)	4,720 (2,830–6,190)	1,550 (1,130–2,010)	3,680 (2,630–4,840)
ANGOLA	1,740 (1,150–2,390)	7,190 (3,940–10,800)	5,140 (3,250–7,530)	5,000 (2,990–7,350)
ALBANIA	33 (17–50)	3 (1–5)	57 (39–81)	73 (38–117)
ARMENIA	70 (43–104)	NA	75 (46–111)	107 (58–175)

CUMULATIVE DEATHS AVERTED BY COUNTRY ACROSS 2030–2100

COUNTRY	ANAL	OROPHARYNGEAL	VAGINAL	VULVAR
**AZERBAIJAN**	529 (309–784)	32 (7–61)	28 (13–44)	394 (243–537)
**BURUNDI**	2,210 (1,610–2,840)	98 (26–194)	1,070 (750–1,450)	4,380 (2,880–6,150)
**BENIN**	1,370 (1,020–1,700)	1,500 (745–2,080)	1,190 (868–1,500)	2,320 (1,690–3,080)
**BURKINA FASO**	9,910 (6,410–13,600)	NA	1,840 (491–3,370)	2,990 (1,590–4,550)
**BANGLADESH**	3,500 (2,600–4,450)	2,220 (707–4,070)	4,560 (3,300–6,090)	9,400 (6,920–12,000)
**BOSNIA AND HERZEGOVINA**	41 (24–60)	65 (32–100)	54 (37–72)	45 (13–87)
**BELARUS**	430 (302–559)	42 (12–76)	125 (87–168)	276 (79–543)
**BELIZE**	NA	NA	NA	NA
**BOLIVIA, PLURINATIONAL STATE OF**	326 (201–472)	1,270 (407–2,580)	991 (525–1,460)	1,010 (533–1,440)
**BHUTAN**	NA	213 (120–338)	NA	NA
**CENTRAL AFRICAN REPUBLIC**	878 (621–1,150)	NA	293 (79–511)	1,370 (925–1,900)
**CHINA**	14,300 (11,300–18,200)	9,140 (4,120–13,500)	13,500 (9,810–17,600)	20,500 (15,200–27,300)
**COTE D'IVOIRE**	4,730 (3,410–5,980)	1,690 (850–2,650)	3,970 (2,650–5,480)	2,980 (2,000–4,080)
**CAMEROON**	3,430 (2,410–4,460)	333 (108–614)	2,250 (1,510–2,980)	3,860 (2,730–5,230)
**CONGO, THE DEMOCRATIC REPUBLIC OF THE**	26,100 (20,100–32,600)	325 (91–587)	8,600 (5,950–11,300)	34,600 (24,500–46,600)
**CONGO**	939 (649–1,240)	155 (49–280)	314 (211–425)	1,190 (832–1,640)
**COLOMBIA**	6,640 (5,790–7,570)	475 (134–797)	2,450 (1,970–2,920)	5,790 (4,040–7,710)
**COMOROS**	NA	NA	NA	178 (112–265)
**CABO VERDE (CAPE VERDE)**	30 (7–50)	NA	NA	23 (6–41)
**CUBA**	664 (450–858)	40 (12–75)	221 (151–298)	513 (352–727)
**DJIBOUTI**	NA	NA	43 (21–66)	123 (82–167)
**DOMINICA**	NA	1 (0–3)	6 (2–10)	1 (0–2)
**ALGERIA**	1,870 (1,370–2,340)	513 (303–702)	1,120 (853–1,420)	2,210 (1,490–3,040)
**ECUADOR**	3,050 (1,990–4,170)	185 (64–330)	768 (542–1,020)	2,550 (1,810–3,430)
**EGYPT**	4,620 (3,600–5,740)	696 (266–1,260)	2,880 (2,070–3,810)	8,710 (6,130–11,500)

CUMULATIVE DEATHS AVERTED BY COUNTRY ACROSS 2030–2100

COUNTRY	ANAL	OROPHARYNGEAL	VAGINAL	VULVAR
**ERITREA**	263 (152–393)	NA	277 (168–407)	632 (450–910)
**ETHIOPIA**	19,900 (14,300–25,300)	3,420 (2,000–5,070)	17,600 (12,800–22,900)	35,400 (24,800–46,900)
**FIJI**	204 (116–282)	7 (1–14)	47 (20–73)	NA
**MICRONESIA, FEDERATED STATES OF**	NA	NA	NA	NA
**GEORGIA**	43 (25–59)	8 (3–16)	61 (41–84)	168 (69–283)
**GHANA**	5,550 (3,520–7,860)	232 (55–516)	994 (606–1,480)	4,200 (2,970–5,580)
**GUINEA**	734 (539–964)	210 (89–356)	1,160 (790–1,520)	554 (362–779)
**GAMBIA**	NA	NA	NA	113 (32–194)
**GUINEA-BISSAU**	NA	NA	52 (14–90)	37 (12–66)
**GRENADA**	12 (8–16)	2 (0–4)	4 (2–6)	10 (5–14)
**GUATEMALA**	312 (167–431)	790 (384–1,220)	554 (344–761)	598 (381–857)
**GUYANA**	20 (5–35)	NA	NA	53 (28–83)
**HONDURAS**	1,360 (925–1,930)	291 (171–422)	754 (465–1,020)	1,090 (766–1,440)
**HAITI**	416 (184–635)	781 (449–1,130)	568 (377–784)	98 (48–155)
**INDONESIA**	6,490 (4,790–8,730)	9,310 (5,810–12,600)	5,910 (4,320–8,250)	21,900 (16,500–28,400)
**INDIA**	65,900 (51,200–84,000)	87,000 (54,700–117,000)	73,000 (52,400–98,900)	81,200 (60,000–104,000)
**IRAN, ISLAMIC REPUBLIC OF**	2,070 (1,820–2,350)	973 (445–1,390)	914 (726–1,120)	873 (665–1,110)
**IRAQ**	1,770 (1,350–2,220)	404 (189–607)	1,190 (814–1,540)	2,170 (1,710–2,760)
**JAMAICA**	77 (57–100)	5 (1–11)	41 (28–59)	72 (49–97)
**JORDAN**	586 (422–770)	174 (91–273)	507 (373–669)	608 (439–799)
**KENYA**	10,700 (7,600–13,900)	1,440 (887–2,070)	2,860 (2,120–3,720)	7,870 (5,670–10,900)
**KYRGYZSTAN**	326 (226–430)	150 (74–253)	249 (120–377)	730 (483–1,110)
**CAMBODIA**	488 (316–674)	158 (65–284)	219 (155–284)	1,490 (922–2,020)
**KIRIBATI**	4 (3–6)	12 (7–19)	1 (0–4)	6 (2–9)
**LAO PEOPLE'S DEMOCRATIC REPUBLIC**	395 (270–493)	457 (273–640)	272 (182–363)	751 (539–994)
**LIBERIA**	356 (257–446)	13 (3–30)	384 (275–507)	287 (181–408)
**ST LUCIA**	NA	NA	NA	NA
**SRI LANKA**	2,280 (1,610–3,050)	129 (37–231)	533 (370–702)	1,760 (1,030–2,430)
**LESOTHO**	240 (160–327)	NA	103 (55–154)	359 (99–639)

CUMULATIVE DEATHS AVERTED BY COUNTRY ACROSS 2030–2100

COUNTRY	ANAL	OROPHARYNGEAL	VAGINAL	VULVAR
MOROCCO	2,120 (1,460–2,700)	121 (29–204)	983 (741–1,280)	2,930 (2,090–3,810)
MOLDOVA, REPUBLIC OF	94 (67–119)	9 (3–16)	17 (10–25)	138 (48–246)
MADAGASCAR	5,830 (4,380–7,460)	206 (59–406)	2,650 (1,900–3,480)	10,300 (6,280–15,100)
MALDIVES	NA	NA	NA	NA
MARSHALL ISLANDS	NA	NA	NA	NA
MACEDONIA (NORTH), THE FORMER YUGOSLAV REPUBLIC OF	106 (74–134)	24 (12–38)	41 (19–66)	55 (28–95)
MALI	4,280 (3,240–5,380)	2,430 (1,350–3,410)	4,010 (3,090–5,030)	5,020 (3,600–6,690)
MYANMAR	1,490 (743–2,160)	240 (81–402)	762 (431–1,180)	2,880 (2,080–3,810)
MONGOLIA	84 (43–127)	21 (6–39)	183 (127–249)	211 (131–301)
MOZAMBIQUE	6,480 (4,650–8,620)	127 (26–309)	3,290 (1,840–4,850)	14,800 (8,570–22,700)
MAURITANIA	739 (577–975)	205 (99–343)	488 (362–636)	7,890 (4,590–11,600)
MALAWI	4,440 (3,340–5,760)	NA	3,840 (2,840–5,070)	4,810 (3,140–6,810)
NAMIBIA	302 (208–402)	43 (10–82)	278 (195–370)	543 (315–800)
NIGER	5,200 (3,430–7,050)	1,700 (782–2,610)	3,760 (2,780–5,010)	1,820 (1,200–2,540)
NIGERIA	28,100 (20,700–36,700)	8,460 (2,880–15,400)	11,900 (7,510–17,300)	47,900 (30,700–66,600)
NICARAGUA	522 (373–688)	277 (156–394)	206 (139–280)	353 (206–519)
NEPAL	628 (421–838)	391 (202–584)	663 (460–892)	729 (490–1,010)
PAKISTAN	15,300 (12,000–19,200)	15,600 (10,800–21,100)	12,900 (9,260–17,200)	15,900 (11,300–21,400)
PERU	1,190 (902–1,500)	1,630 (917–2,250)	1,160 (861–1,500)	1,220 (836–1,680)
PHILIPPINES	2,930 (2,110–3,740)	733 (232–1,330)	2,180 (1,470–2,900)	5,900 (4,440–7,570)
PAPUA NEW GUINEA	1,710 (1,100–2,340)	297 (69–554)	511 (244–781)	739 (463–1,050)
KOREA, DEMOCRATIC PEOPLE'S REPUBLIC OF	233 (170–300)	129 (68–183)	285 (182–409)	616 (448–811)
PARAGUAY	1,450 (1,050–1,770)	273 (128–444)	30 (9–50)	981 (671–1,480)

CUMULATIVE DEATHS AVERTED BY COUNTRY ACROSS 2030–2100

COUNTRY	ANAL	OROPHARYNGEAL	VAGINAL	VULVAR
PALESTINE, STATE OF	365 (284–446)	274 (154–395)	206 (149–271)	549 (392–733)
RWANDA	1,650 (1,220–2,110)	38 (13–67)	839 (617–1,080)	4,610 (3,400–6,210)
SUDAN	3,770 (1,850–5,790)	1,060 (609–1,600)	3,380 (1,010–5,890)	8,930 (5,900–12,200)
SENEGAL	152 (44–273)	NA	NA	NA
SOLOMON ISLANDS	162 (113–212)	11 (2–18)	91 (63–123)	346 (233–478)
SIERRA LEONE	1,010 (745–1,310)	22 (6–39)	639 (473–837)	1,750 (1,270–2,250)
EL SALVADOR	274 (199–346)	45 (20–73)	51 (29–74)	118 (71–172)
SOMALIA	2,570 (1,840–3,310)	116 (31–230)	939 (631–1,290)	3,880 (2,740–5,240)
SERBIA	660 (252–1,030)	NA	190 (54–339)	NA
SOUTH SUDAN	NA	414 (99–960)	1,300 (379–2,280)	3,210 (1,030–5,580)
SAO TOME AND PRINCIPE	11 (7–15)	6 (2–11)	6 (3–9)	7 (1–13)
SWAZILAND	438 (277–601)	NA	182 (99–265)	251 (67–452)
SYRIAN ARAB REPUBLIC	689 (495–934)	297 (165–455)	525 (354–700)	1,550 (1,050–2,180)
CHAD	NA	185 (44–404)	1,400 (565–2,400)	3,800 (1,550–5,900)
TOGO	662 (497–847)	168 (52–299)	908 (606–1,310)	1,230 (849–1,610)
THAILAND	1,230 (955–1,550)	233 (71–422)	856 (625–1,100)	2,190 (1,610–2,760)
TAJIKISTAN	1,010 (722–1,370)	488 (289–725)	41 (13–72)	518 (358–723)
TURKMENISTAN	878 (643–1,130)	651 (397–957)	334 (223–468)	695 (452–982)
TIMOR-LESTE	NA	NA	NA	NA
TONGA	2 (1–4)	NA	2 (1–5)	4 (2–7)
TUNISIA	709 (558–861)	110 (54–167)	315 (236–406)	772 (566–1,010)
TUVALU	NA	NA	NA	NA
TANZANIA, UNITED REPUBLIC OF	21,400 (15,200–28,300)	3,800 (1,080–6,830)	9,660 (7,040–12,500)	22,500 (14,500–33,000)
UGANDA	4,540 (3,190–5,820)	2,690 (1,230–4,130)	3,980 (2,770–5,290)	13,000 (8,500–18,300)
UKRAINE	1,130 (751–1,450)	1,210 (675–1,710)	558 (396–712)	874 (273–1,540)
UZBEKISTAN	4,750 (3,410–6,280)	2,940 (1,840–3,890)	1,290 (921–1,750)	2,970 (1,960–4,080)
ST VINCENT & THE GRENADINES	NA	NA	NA	NA

CUMULATIVE DEATHS AVERTED BY COUNTRY ACROSS 2030–2100

COUNTRY	ANAL	OROPHARYNGEAL	VAGINAL	VULVAR
VENEZUELA, BOLIVARIAN REPUBLIC OF	2,920 (1,940–4,010)	1,170 (675–1,660)	1,530 (1,030–2,020)	1,180 (793–1,580)
VIET NAM	4,170 (3,230–5,220)	2,150 (1,360–2,840)	777 (564–1,050)	2,790 (1,990–3,740)
VANUATU	92 (61–125)	NA	NA	NA
SAMOA	17 (13–21)	1 (0–2)	12 (7–18)	22 (17–29)
KOSOVO	89 (68–113)	6 (2–11)	44 (30–60)	141 (71–217)
YEMEN	640 (297–992)	547 (189–1,070)	2,290 (1,570–3,120)	1,770 (1,170–2,350)
SOUTH AFRICA	2,390 (1,610–3,160)	1,630 (914–2,330)	2,060 (1,460–2,830)	1,450 (967–1,990)
ZAMBIA	4,950 (3,660–6,270)	88 (28–159)	3,720 (2,430–5,320)	7,750 (4,940–11,200)
ZIMBABWE	2,690 (1,990–3,610)	112 (33–203)	2,020 (1,370–2,770)	6,050 (3,880–8,860)

Table S8: Country-wise impact of HPV vaccination on cancer outcomes (2030–2100).

Note: 95% uncertainty interval in parentheses

lb= lower bound; NA= not applicable; ub= upper bound.
